# Chemical constituents and anti-inflammatory activities of Maqian (*Zanthoxylum myriacanthum* var. *pubescens*) bark extracts

**DOI:** 10.1038/srep45805

**Published:** 2017-04-06

**Authors:** Huan-li Zhang, Xiao-qing Gan, Qing-fei Fan, Jing-jing Yang, Ping Zhang, Hua-bin Hu, Qi-shi Song

**Affiliations:** 1 Key Laboratory of Tropical Plant Resources and Sustainable Use, Xishuangbanna Tropical Botanical Garden, Chinese Academy of Sciences, 88 Xuefu Road, Kunming, Yunnan 650223, PR China; 2 Department of Life Sciences, University of Chinese Academy of Sciences, 19A Yuquan Road, Beijing 100049, PR China

## Abstract

In this study, 44 compounds in the petroleum ether extract of Maqian (*Zanthoxylum myriacanthum* var. *pubescens*) bark, a traditional Dai herbal medicine, were identified by GC-MS. Major components included 3(2H)-benzofuranone, asarinin and (dimethoxymethyl)-3-methoxy-benzene. A total of 18 compounds were isolated from the ethyl acetate extracts of Maqian bark by column chromatography and identified by chemical and spectral analyses. Rhoifoline B, zanthoxyline dimethoxy derivative, N-nortidine, nitidine, decarine are the major alkaloids. Both the petroleum ether and ethyl acetate extracts showed significant inhibition on NO production, which imply anti-inflammatory activity, in lipopolysaccharide-induced RAW 264.7 cells without cell toxicity. Decarine is the major anti-inflammatory constituent with NO IC_50_ values of 48.43 μM on RAW264.7 cells. The petroleum ether extract, the ethyl acetate extract and decarine showed anti-inflammatory activities through inhibiting TNF-α and IL-1β production in lipopolysaccharide-stimulated THP-1 cells without cell toxicity too. Decarine showed anti-inflammatory activity on human colon cells by reducing IL-6 and IL-8 production in TNF-α+IL-1β-induced Caco-2 cells. These results support the use of Maqian bark as a remedy for enteritis and colitis recorded by Dai medicine in China, and elucidate the major pharmacological compounds in Maqian bark.

Maqian (*Zanthoxylum myriacanthum* var. *pubescens* Huang (Huang)) is a valuable traditional Dai herb medicine found in the south of Yunnan province of China. Dai people use Maqian bark as a home remedy for enteritis, pediatric hepatitis and colitis^1^. Maqian is not only a Dai herb medicine with detoxification, swelling reduction and pain relief effects, but is also used to flavor cooking sauces in the Dai cuisine^2^.

Maqian is a variation of *Zanthoxylum myriacanthum*, which is widely used as food flavoring in China^3^. Its branch, leaflet and fruits have a strong aroma of pepper or another distinctive smell arising from many tiny oil glands^4^. The fruit peels of *Z. myriacanthum* contain essential oils with high levels of monoterpene hydrocarbons and oxygenated monoterpenes. The essential oils also contain *n-*alcohols and sesquiterpenes which demonstrate relatively strong anti-microbial activities^5^. The inner bark of *Z. myriacanthum* is rich in sulfur-like amyloid cylinder. The bark and leaves are used as spicy and bitter tasting herbs which have the medicinal effects of anti-rheumatism, blood pressure stabilizing, swelling and pain relief^6^. Studies have shown that roots and stem bark of this plant genus are rich in triterpenoids, lignans^7^ and alkaloids^8^. Extracts of leaves, stems and bark are reported to have anti-tumor^9^ and anti-inflammatory activities^10^.

One study reported the chemical composition and bioactivities of Maqian fruits. That study indicated that the essential oil of Maqian fruits was rich in limonene, and had significant inhibitory and lethal effects on common food-borne pathogens or microbes which induce food spoilage^11^.

Nitric oxide (NO) is an endogenous free radical. Normally, NO content is low in the human body. Its physiological functions are vasodilatation, neuro signal transduction, non-specific host defense and immunoreaction mainly as a signal molecule^12^. Inhibition of NO production was usually used as a pharmacologically important treatment of acute and chronic inflammation-related diseases. Whether a compound inhibits NO production is indicative of whether the compound has anti-inflammatory activity.

The macrophage cell is the key immune cell in the initial period of inflammation^13^. Bacterial endotoxins like lipopolysaccharide (LPS) induce the macrophage cell’s inflammatory reaction^14^. LPS-induced RAW 264.7 macrophages could produce large amounts of NO. This could be used to assess the anti-inflammatory activities of samples.

A series of inflammatory reaction including synthesis and release of some relevant cytokine were occur after LPS induced normal cells. TNF-α is a key pro-inflammatory cytokine. LPS could induce TNF-α synthesis and promote the expression of some relevant cytokine, chemokine and endothelial cell adhesion molecule^15^. IL-1β, another pro-inflammatory cytokine synthesized by macrophage in the early inflammation, could induce IL-6 synthesis and more IL-1β production^16^. IL-1β and TNF-α stimulate inflammatory responses in human colon cancer cells (Caco-2) by inducing the production of IL-6 and IL-8^17^, which could be used to evaluate the anti-inflammatory activities of samples on intestinal cells further.

As a part of our ongoing search for bioactive secondary metabolites from Chinese tropical medicinal plants, a careful investigation on the chemical constituents of Maqian bark, led to 44 components being identified in the petroleum ether extract by GC-MS. In addition, 18 compounds were isolated and identified from the ethyl acetate extract. Moreover, the anti-inflammatory activities of the extracts and compounds by reducing inflammatory factors (NO, TNF-α and IL-1β) production in LPS-induced murine and human macrophages inflammation were studied, and the petroleum ether extract and ethyl acetate extract showed anti-inflammatory activity among the four kinds of extracts (crude, petroleum ether, ethyl acetate and *n*-butanol) being tested. Anti-inflammatory activities of the ethyl acetate extract are mainly due to the existence of decarine.

## Materials and Methods

### Plant materials

Maqian (*Zanthoxylum myriacanthum* var. *pubescens*) bark was collected from Mengwang township, Jinghong City, Xishuangbanna Prefecture, China in August 2014, and identified by Chun-fen Xiao of Xishuangbanna Tropical Botanical Garden, Chinese Academy of Science. A voucher specimen (no.152673) of Maqian was placed in the herbarium (HITBC).

### Extraction, isolation and identification

The samples were air dried and powdered. The powdered Maqian bark (18.0 kg) was extracted 3 times with 90% methanol in a hot water bath to produce the crude extract (2499 g). The water suspension of the condensed crude extract was successively extracted with petroleum ether, ethyl acetate and *n*-butanol for 4–5 times each. Following the solvent removal, the petroleum ether (15 g), ethyl acetate (473 g) and *n*-butanol (975 g) extracts were obtained. A 2 g portion of the petroleum ether extract was submitted for GC-MS chemical composition analysis in the Plant Chemical Analysis and Testing Center of Kunming Institute of Botany.

The ethyl acetate extract residue was loaded onto silica gel (200–300 mesh) and eluted with a chloroform (C)/methanol (M) gradient (100% C – 1:1) to produce seven fractions (1–7) based on TLC analysis. Filtration of the crystalline solid from fraction 1 (40 g) yielded compounds **15** (75 mg) and **16** (60 mg). Compound **1** (6 mg) was obtained by Sephadex LH-20 eluted with C/M (1:1) from 1 g of fraction 1. Fraction 2 (65 g) was separated into four sub-fractions (2A-2D) by silica gel (200–300 mesh) eluted with a petroleum ether (P)/ acetone (A) gradient (20:1–2:1). Filtration of the crystalline solid from sub-fraction 2D (3 g) yielded **12** (45 mg) and **18** (45 mg). Sub-fractions 2 A (90 mg) and 2 C (70 mg) were purified by Sephadex LH-20 eluted with C/M (1:1) to yield **3** (15 mg) and **11** (5 mg) respectively. Filtration of the crystalline solid from fraction 3 (45 g) yielded **17** (35 mg). The remaining portion (21 g) of fraction 3 was separated into six sub-fractions (3A-3F) by silica gel (200–300 mesh) eluted with a C/M gradient (50:1–4:1). Sub-fraction 3 A (90 mg), 3 C (70 mg) and 3D (46 mg) were purified by Sephadex LH-20 eluted with C/M (1:1) to yield **4** (2 mg), **5** (8 mg) and **6** (6 mg) respectively. Fraction 4 (30 g) was separated into five sub-fractions (4A-4E) by silica gel (200–300 mesh) eluted with a C/M gradient (20:1–1:1). Filtration of the crystalline solid from sub-fraction 4D (2 g) yielded **2** (20 mg). Sub-fraction 4 A (50 mg) and 4B (85 mg) were additionally purified by Sephadex LH-20 eluted with C/M (1:1) to yield **13** (5 mg) and **14** (10 mg) respectively. A portion (2 g) of fraction 5 was separated into four sub-fractions (5A-5D) by Sephadex LH-20 eluted with C/M (1:1). Sub-fraction 5 A (50 mg) and 5 C (120 mg) were purified by Sephadex LH-20 eluted with C/M (1:1) to yield **7** (8 mg) and **10** (30 mg) respectively. Filtration of the crystalline solid from fraction 6 (20 g) yielded **8** (20 mg). A portion (3 g) of fraction 6 was purified by Sephadex LH-20 eluted with C/M (1:1) to yield **9** (2 mg) and **10** (20 mg).

Compounds were identified by chemical and spectral analyses. ^1^H and ^13^C NMR spectra were obtained on a Bruker-DRX-500 spectrometer with chemical shifts recorded in *δ* (ppm) using tetramethylsilane (TMS) as the internal standard, while the coupling constants (*J*) were given in hertz. Mass spectra were obtained on a MS Waters AutoSpec Premier P776 mass spectrometer (EI-MS) and a Micro Q-TOF MS (HERSIMS), respectively.

### GC–MS analysis

Analysis of the chemical constituents of petroleum ether extract of Maqian bark was done with an Agilent Technologies HP6890GC/5973MS.

GC condition: HP-5MS capillary column (30 mm’ 0.25 mm’ 0.25 mm); column temperature: initial temperature of 40 °C, temperature-programmed 3 °C/min to 80 °C, then programmed temperature 5 °C/min to 260 °C maintain 60 min; column flow rate 1.0 ml/min; inlet temperature 250 °C; pre-column pressure 100 kPa; split ratio of 10: 1; injection volume 2.0 ml; high purity helium as the carrier gas.

MS condition: ionization mode EI; electron energy 70; transmission line temperature 250 °C; ion source temperature was 230 °C; quadrupole temperature 150 °C; mass range of 35 to 500; using wiley7n.l standard library searching for qualitative.

### Cells and culture

Murine macrophage cells RAW 264.7 were purchased from Kunming Institute of Zoology, Chinese Academy of Sciences (KCB200603YJ) and cultured in Dulbecco’s modified Eagle’s medium (DMEM, Thermo Scientific, Logan, UT, USA) containing 10% fetal bovine serum (FBS), 1% penicillin-streptomycin and 1% L-glutamine (Sigma-Aldrich, St Louis, MO, USA) at 37 °C in a 5% CO_2_ incubator (Thermo Scientific, Forma 371, Steri-cycle, USA) and sub-cultured every 2 days.

Human monocytic THP-1 cells were obtained from Conservation Genetics CAS Kunming Cell Bank (ATCC Number: TIB-202) (Kunming, Yunnan, China), and maintained in RPMI 1640 medium supplemented with 10% fetal bovine serum and 1% penicillin-streptomycin. Cells were cultured in a humidified CO_2_ incubator at 37 °C with 5% CO_2_. Media were changed once every 72 h. In all experiments, THP-1 cells were incubated in the presence or absence of various concentrations of drugs that were always added 30 min prior to lipopolysaccharide (1 μg/ml) treatment.

Human colon cancer cell line Caco-2 was obtained from Kunming Institute of Zoology, Chinese Academy of Sciences and cultured at 37 °C under 5% CO_2_ atmosphere using Dulbecco’s Modified Eagle’s Medium (Gibco, Grand Island, USA) supplemented with 15% (v/v) FBS (Gibco, Grand Island, USA).

### Chemicals and reagents

Dimethylsulfoxide (DMSO), lipopolysaccharide (LPS) and dexamethasone (Dex) were bought from Sigma-Aldrich (St. Louis, MO, USA). Dulbecco’s modified Eagle’s medium, fetal bovine serum and penicillin–streptomycin were obtained from Thermo Scientific (Logan, UT, USA). CellTiter 96 A_Queous_ One Solution Reagent for MTS assay and Griess reagent system for NO measurement were obtained from Promega Corporation (Madison, WI, USA). Standard Mueller–Hinton agar and broth (MHA, MHB) and Sabouraud agar and broth (SA, SB) were purchased from Tianhe Microbial Agents Company (Hangzhou, China). Human IL-1β, TNF-α ELISA kit were bought from BD Biosciences San Diego (CA, USA). All reagents were analytical standard.

### Cell viability test

Cell viability was measured by MTS assay^18^. In the MTS assay, 100 μl cell suspensions (1 × 10^6^ cells/ml) were cultured in 96-cell plates for 18 h. Then cells were pre-treated with four concentration gradients of the four extracts, two compounds and 10 μM Dex for 30 min before they were further incubated in the presence of 1 μg/ml lipopolysaccharide for 24 h. 20 μl of CellTiter 96 A_Queous_ One Solution Reagent, prepared by MTS (3-[4,5,dimethylthiazol-2-yl]-5-[3-carboxymethoxy-phenyl]-2-[4-sulfophenyl]-2H-tetrazolium, inner salt) in phenazine ethosulfate, was put to each well and incubated for 1 h at 37 °C in the 5% CO_2_ incubator. Absorbance of each well was measured at 490 nm applying a Multi-functional Microplate Reader (Thermo Scientific VarioSkan Flash, USA). The untreated cells, incubated in medium with 0.4% dimethylsulfoxide, were used as blanks in every test. Results were presented as a percentage of the untreated control cells. Values were stated as mean ± standard deviation (SD) of three tests.

### Measurement of NO, TNF-α and IL-1β production

Nitric oxide (NO) plays a key role in inflammation and carcinogenesis^19^. This study used NO production of lipopolysaccharide-stimulated RAW 264.7 cells as inflammation contrast, Maqian extracts and compounds treated lipopolysaccharide-stimulated RAW264.7 cells and inhibited NO release at different concentration gradients to show the anti-inflammatory activity. Anti-inflammatory drug dexamethasone (Dex) was used as positive contrast.

Cells were set in the 96-well plates at 1 × 10^5^ cells/well and incubated for 18 h. Then cells were pretreated with gradient concentrations of the extracts and 10 μM Dex for 30 min before they were stimulated with 1 μg/ml lipopolysaccharide for 24 h. 50 μl of culture supernatants were taken and blended with the Griess reagent. NO production by lipopolysaccharide-stimulated RAW 264.7 cells was measured by the Griess reagent system.

THP-1 cells (1 × 10^6^ cells/ml) were treated with or without LPS (1 μg/ml) after 30 min of pre-exposure to the extracts (10, 20, 40 or 80 μg/ml, respectively) or Dex (5 μM). After 24 h, supernatants were collected and the concentrations of TNF-α and IL-1β were measured using their corresponding ELISA kits. Cells were first treated with PMA for 24 h to differentiate into macrophages. Then the cells were pretreated with the extracts or Dex followed by culturing with LPS for 24 h. Supernatant was harvested for determination of NO by the Griess reagent method.

### Measurement of IL-6 and IL-8 production

Caco-2 cells were seeded at a density of 1.5 × 10^4^ cells/well in 96-well plates. 24 h after seeding, the cells were then washed with phosphate buffered saline (PBS, 137 mM NaCl, 2.68 mM KCl, 1.14 mM KH_2_PO_4_, 8 mM Na_2_HPO_4_, pH 7.2), and cells were exposured to IL-1β (25 ng/ml) and TNF-α(50 ng/ml) (Peprotech, Rocky Hill, USA) to allow inflammatory responses. Dexamethasone (Sigma-Aldrich, St. Louis, USA) at 5 μM was used as control. Each treatment was applied for 24 h in culture medium containing 1% (v/v) FBS. The extracellular media were collected and centrifuged at 3000 rpm for 20 min. IL-6 and IL-8 secretion was evaluated using ELISA kits (Dakewe, Shenzhen, CN) according to manual instruction.

### Statistical analysis

One Way ANOVA and Dunnett Multiple Comparison tests was used to check the significance of the cell viability difference between the treatments and the blank, the NO, TNF-α and IL-1β production differences between Maqian extracts (compounds) treated LPS-stimulated cells and LPS-stimulated cells, and the IL-6 and IL-8 production differences between decarine treated (TNF-α+IL-1β)-stimulated cells and (TNF-α+IL-1β)-stimulated cells.

## Results

### Chemical composition of the petroleum ether and ethyl acetate extract

**Figure 1:**
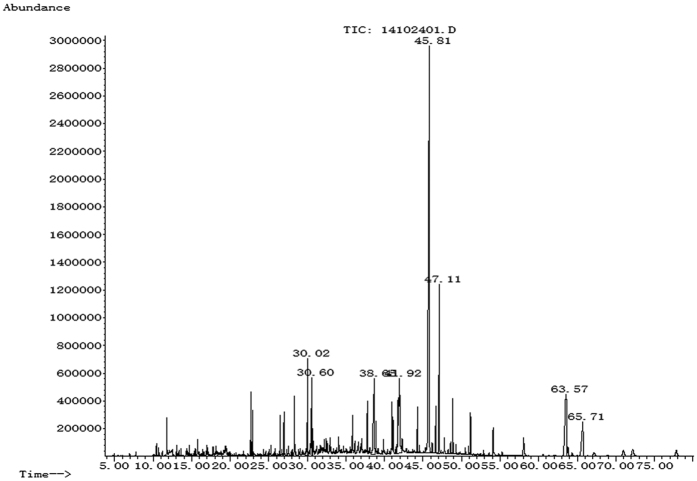
The total ion chromatogram (TIC) of GC-MS of petroleum ether extract from *Zanthoxylum myriacanthum* var. *pubescens* bark.

Forty four compounds (Table 1, Fig. 1) were identified in the petroleum ether extract, representing 75.542% of the petroleum ether extract of Maqian bark. Among them, 3(2H)-benzofuranone (26.173%), asarinin (6.484%), (dimethoxymethyl)-3-methoxy-benzene (5.443%), hexadecanoic acid (4.378%), 9,12-octadecadienoic acid (4.119%), nerolidol (2.451%), 9-octadecenoic acid (2.138%), (−)-caryophyllene oxide (2.023%), 2-tridecanone (1.349%), bornyl acetate (1.250%), (+)-spathulenol (1.246%), hexadecanoic acid, methyl ester (1.133%), were the major compounds, which accounted for 58.187% of the total compounds in the extract.

Compounds **1–18** (Table 2) were isolated from the ethyl acetate extract of Maqian bark. Compounds **1–14** and **18** were identified by comparison of their spectroscopic data with data reported in the literature. Compounds **15–17** were identified by comparison with TLC Rf value of the corresponding standard substance. Compounds **2, 8, 10, 12, 15, 16, 17,** and **18** were the major constituents in the ethyl acetate extract of Maqian bark. Compounds **11, 12, 13, 14,** and **18** were alkaloids. Compounds **1–14** and **18** were obtained from this species for the first time. Compounds **1, 3, 8, 10,** and **12** were obtained from this genus for the first time.

### Cell viability

**Figure 2:**
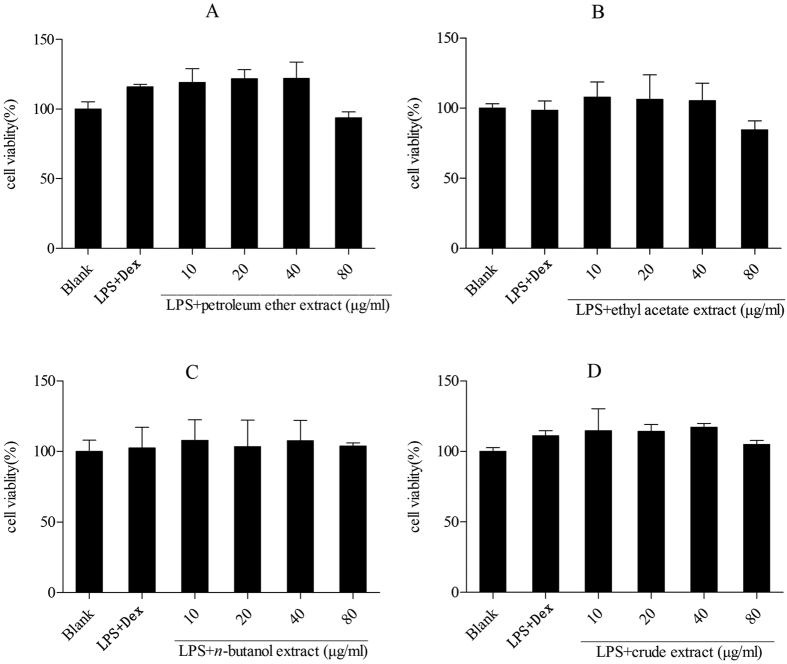
Effect of the extracts of *Zanthoxylum myriacanthum* var. *pubescens* on the cell viability of RAW 264.7 macrophages. All values were means ± SD, n = 3. Dex: dexamethasone.

**Figure 3:**
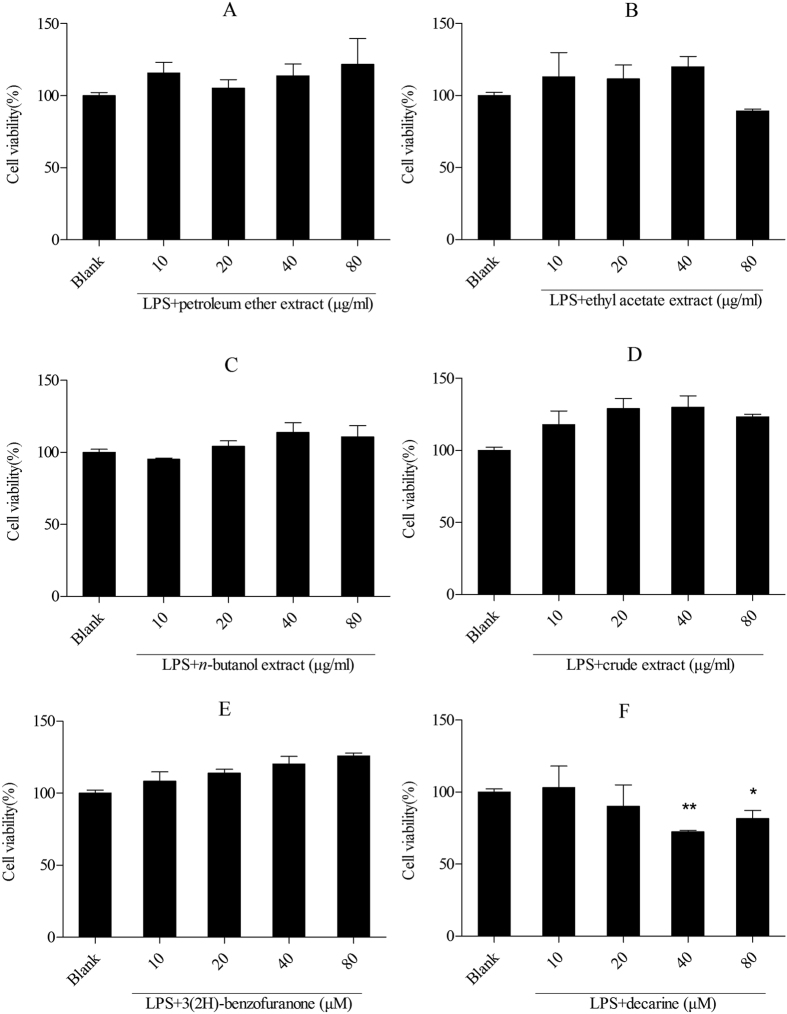
Effect of the extracts and compounds of *Zanthoxylum myriacanthum* var. *pubescens* on the cell viability of THP-1 cells. All values were means ± SD, n = 3.

The effect of the four kinds of Maqian bark extracts on cell viability was determined by the MTS assay. All extracts showed no effects on the cell viability of murine RAW 264.7 macrophages and human THP-1 cells at 10 μg/ml, 20 μg/ml, 40 μg/ml, 80 μg/ml (Figs 2 and 3). The ethyl acetate extract reduced cell viability slightly at 80 μg/ml. However, the viabilities of cells treated with 80 μg/ml ethyl acetate extract were not significantly different from those of the untreated control cells, as tested by Dunnett’s multiple comparison tests, implying that the anti-inflammatory activities of the extracts up to 80 μg/ml were not implicated with cell toxicity. Therefore, we inferred that concentrations up to 80 μg/ml of the extracts could be safe for the development of Maqian bark as a drug.

There was no decrease in cell viability after treatment of 3(2H)-benzofuranone up to 80 μM in THP-1 cells (Fig. 3). Decarine did not reduce viability of THP-1 cells up to the concentration of 20 μM. The results indicated that decarine isolated from the ethyl acetate extract did not exhibit any cytotoxic effect at the employed concentrations of 10 and 20 μM, and 3(2H)-benzofuranone from the petroleum ether extract did not show cytotoxic effect at the employed concentrations of 10, 20, 40 and 80 μM.

**Figure 4:**
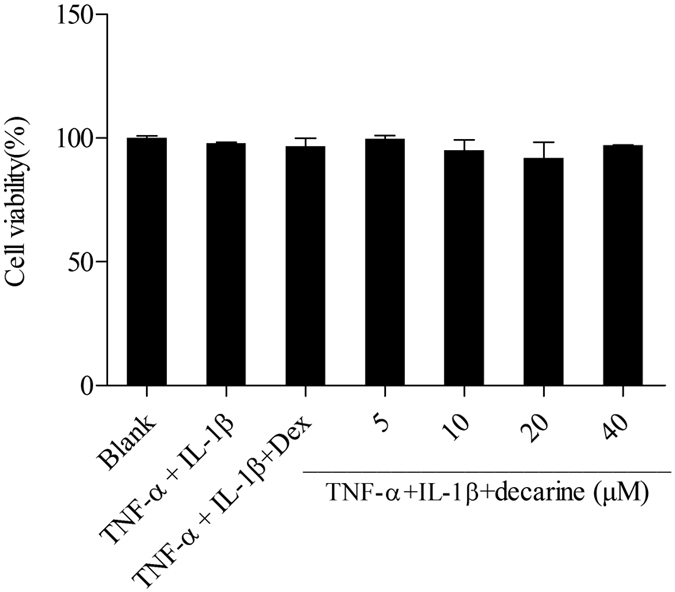
Effect of decarine on the cell viability of Caco-2 cells. All values were means ± SD, n = 3. Dex: dexamethasone; IL-1β: interleukin-1β; TNF-α: tumor necrosis factor-alpha.

Decarine did not reduce viability of Caco-2 cells up to the concentration of 40 μM (Fig. 4). The results indicated that decarine isolated from the ethyl acetate extract of *Zanthoxylum myriacanthum* var. *pubescens* did not exhibit any cytotoxic effect at the employed concentrations of 10. 20 and 40 μM.

### Effect on NO production in lipopolysaccharide-induced RAW 264.7 cells

**Figure 5:**
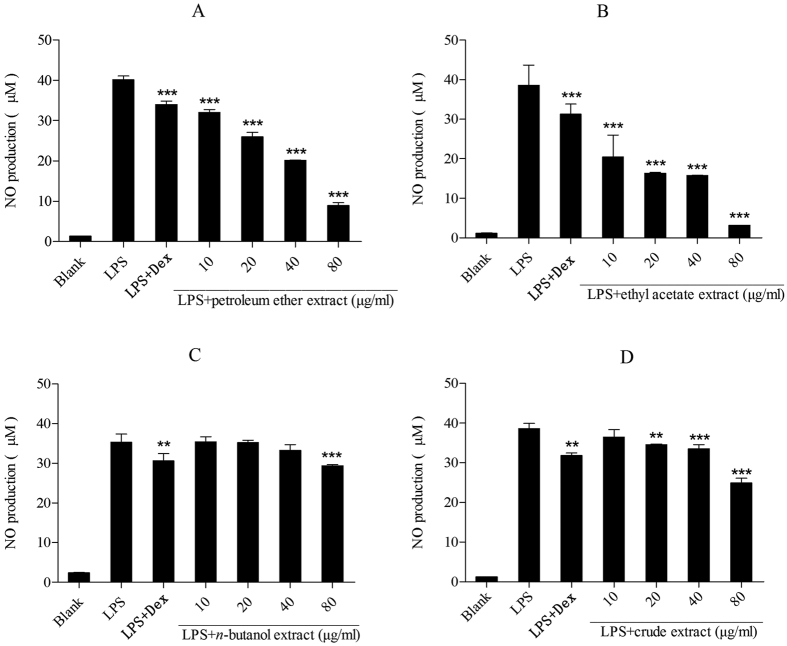
Effect of the extracts and compounds of *Zanthoxylum myriacanthum* var. *pubescens* on NO production in LPS-induced RAW 264.7 cells. All values were means ± SD, n = 3. (*P < 0.05, **P < 0.01, ***P < 0.001) ***P < 0.001 indicated very significant difference with the LPS treated cells. LPS: lipopolysaccharide; Dex: dexamethasone.

The effect of the extracts of Maqian bark on NO production in lipopolysaccharide-induced RAW 264.7 cells was determined by Griess reagent system. Lipopolysaccharide significantly increased NO production compared to untreated cells (“Blank” in Fig. 5). All extracts showed a dose-dependent inhibition of NO production (Fig. 5). The petroleum ether and ethyl acetate extracts showed a dose-dependent inhibition of NO production (Fig. 5A,B). However, the *n*-butanol extract and crude extract showed a quite low dose-dependent inhibition of NO production (Fig. 5C,D).

**Figure 6:**
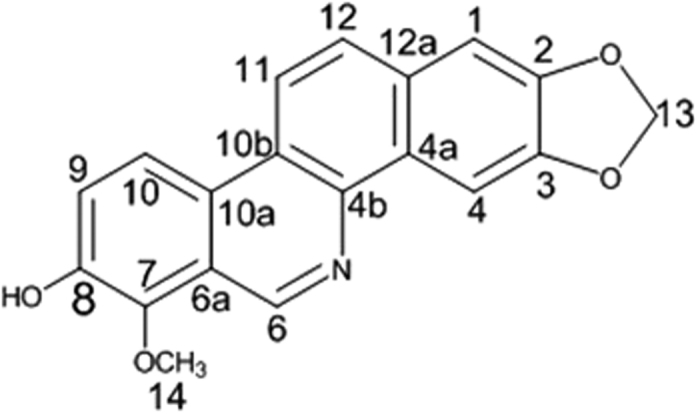
Chemical structure of decarine (18)^34^.

According to the effect of the anti-inflammatory activity of extracts from Maqian bark, the inhibitory activity of the ethyl acetate extract was significantly higher than that of the other two Maqian bark extracts at all tested concentrations, indicating it had a better anti-inflammatory activity. Results showed that the petroleum ether extract also had a significant anti-inflammatory activity while the *n*-butanol extract had only very weak activity. Decarine (**18**) (Fig. 6) is the major anti-inflammatory active constituent in the ethyl acetate extract with IC_50_ values of 48.43 μM (14.67 μg/ml) (Table 3).

Decarine (**18**), an alkaloid, was obtained as a brown, amorphous powder and assigned the molecular formula C_19_H_13_O_4_N on the basis of its ^13^C NMR data and ESI-MS ion at *m/z* 319 [M]^+^. ^1^H NMR (600 MHz, Acetone-*d*_*6*_) *δ* 10.07 (1H, s, 8-OH), 9.62 (1H, s, H-6), 8.51 (1H, s, H-4), 8.50 (1H, d, *J* = 8.0Hz, H-11), 8.47 (1H, d, *J* = 8.0 Hz, H-10), 7.95 (1H, d, *J* = 8.0 Hz, H-12), 7.60 (1H, d, *J* = 8.0 Hz, H-9), 7.40 (1H, s, H-1), 6.19 (2H, s, -O-CH_2_-O-), 3.96 (3H, s, 10-OCH_3_). ^13^C NMR (150 MHz, Acetone-*d*_*6*_) *δ* 148.24 (C-3), 148.02 (C-2), 146.75 (C-9), 145.51 (C-6), 143.18 (C-10), 129.4 (C-12a), 128.22 (C-12), 127.93 (C-6a), 123.90 (C-8), 123.80 (C-10a), 119.31 (C-11), 119.11 (C-7), 105.15 (C-1), 102.44 (C-4), 102.29 (-O-CH_2_-O-), 62.23 (10-OCH_3_). The ^1^H and ^13^C NMR data were similar to those found in the literature for decarine^34^.

### Effect on TNF-α and IL-1β production in lipopolysaccharide-induced THP-1 cells

**Figure 7:**
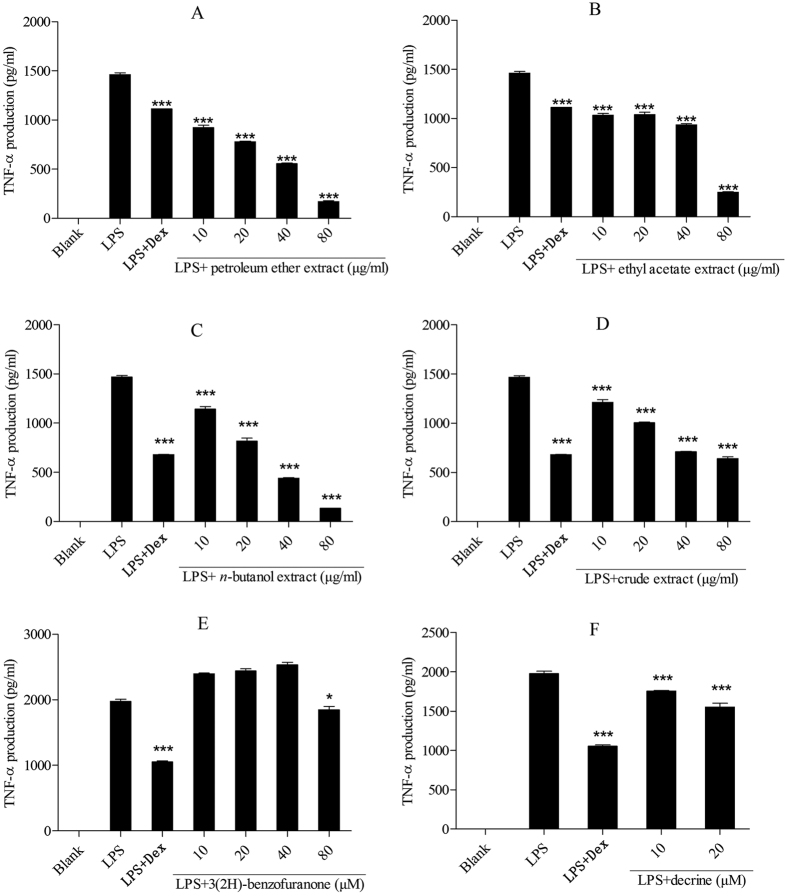
Effect of the extracts and compounds of *Zanthoxylum myriacanthum* var. *pubescens* on TNF-α production in LPS-induced THP-1 cells. All values were means ± SD, n = 3. (*P < 0.05, **P < 0.01, ***P < 0.001) ***P < 0.001 indicated significant difference with the LPS treated cells. LPS: lipopolysaccharide; Dex: dexamethasone; TNF-α: tumor necrosis factor-alpha.

**Figure 8:**
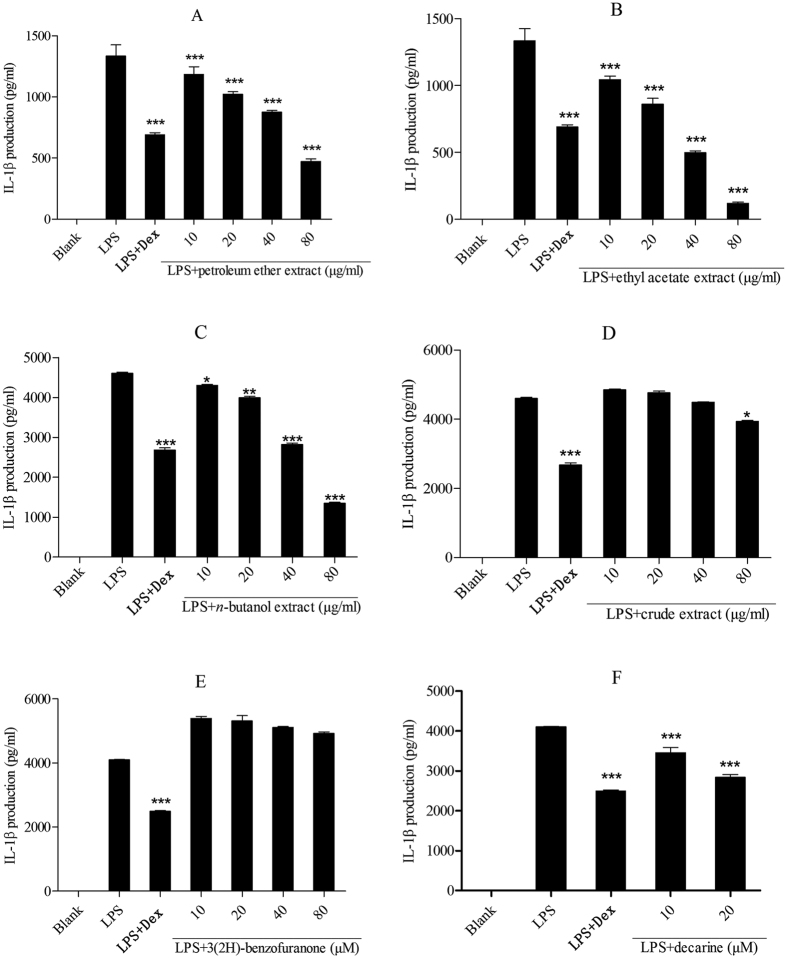
Effect of the extracts and compounds of *Zanthoxylum myriacanthum* var. *pubescens* on IL-1β production in LPS-induced THP-1 cells. All values were means ± SD, n = 3. (*P < 0.05, **P < 0.01, ***P < 0.001) ***P < 0.001 indicated very significant difference with the LPS treated cells. LPS: lipopolysaccharide; Dex: dexamethasone; IL-1β: interleukin-1β.

A series of inflammatory reactions including transcription and synthesis of some relevant cytokines were opened after lipopolysaccharide induced normal cells. All samples significantly inhibited NO release and showed potent anti-inflammatory activities. In order to make sure the samples inhibit cytokine production, the study measured the effect of extracts on TNF-α and IL-1β production in LPS-induced THP-1 cells. The results indicated that the petroleum ether extract, ethyl acetate extract and decarine (**18**) showed a dose-dependent inhibition of TNF-α and IL-1β production (***P < 0.001) and they had a good anti-inflammatory activity (Figs 7A,B,F and 8A,B,F).

The *n*-butanol extract showed significant inhibitions on TNF-α and IL-1β production (*P *<* 0.05, **P < 0.01, ***P < 0.001, Figs 7C and 8C) and indicated a potent anti-inflammatory activity. Crude extract showed a good activity by declining TNF-α production (***P *<* 0.001, Fig. 7D) and significant inhibitions on IL-1β production only at the concentration of 80 μM (*P *<* 0.05, Fig. 8D). Decarine exhibited significant inhibitions on TNF-α and IL-1β production (***P < 0.001, Figs 7F and 8F), which implies that it has anti-inflammatory activity. 3(2H)-benzofuranone showed a weak activity by declining TNF-α production at the concentration of 80 μM (Fig. 7E). However IL-1β production was not significantly inhibited at all concentrations treated by 3(2H)-benzofuranone. That indicates 3(2H)-benzofuranone has no anti-inflammatory activity on IL-1β production in LPS-induced THP-1 cells (Fig. 8E).

**Figure 9:**
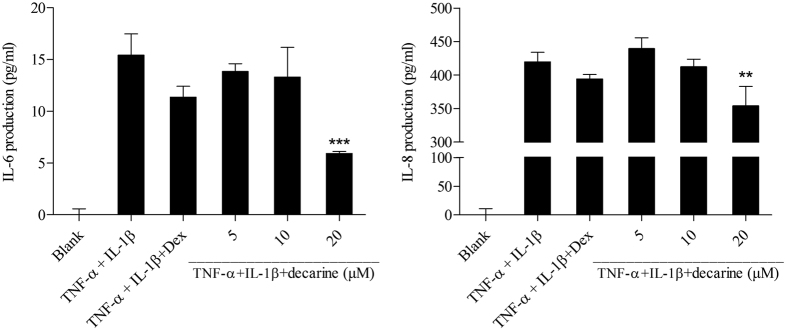
Effect of decarine on IL-6 and IL-8 production in IL-1β+TNF-α-induced Caco-2 cells. All values were means ± SD, n = 3. (*P < 0.05) *P < 0.05 indicated significant difference with cells treated by IL-1β and TNF-α. Dex: dexamethasone; IL-1β: interleukin-1β; TNF-α: tumor necrosis factor-alpha; IL-6: interleukin-6; IL-8: interleukin-8.

Decarine showed significant inhibitions on IL-6 and IL-8 production in TNF-α+IL-1β-induced Caco-2 cells at the concentration of 20 μM (*P < 0.05, Fig. 9), which indicated that it has anti-inflammatory activity on human colon cells. Anti-inflammatory drug dexamethasone showed slight but not significant inhibitions on the IL-6 and IL-8 production at 5 μM, which meant large concentration of dexamethasone might be needed to decrease the IL-6 and IL-8 production.

## Discussion

The petroleum ether and ethyl acetate extracts of Maqian bark showed a dose-dependent inhibition of NO production (Fig. 5A,B), indicating that they have anti-inflammatory activity. The inhibitory activity of the ethyl acetate extract was significantly higher than that of the other two extracts at all tested concentrations, indicating a better anti-inflammatory activity. The *n*-butanol extract and crude extract showed a certain extent of anti-inflammatory activity on inhibiting NO and IL-1β release (Figs 5C,D and 8C,D) but showed a significant inhibition of TNF-α production (Fig. 7C,D). These results are consistent with previous studies that reported extracts from the *Zanthoxylum* were used to treat inflammatory pain^35^.

The extracts and some compounds of Maqian showed significant inhibition of TNF-α and IL-1β production (Figs 7 and 8), which indicates further that Maqian extracts and compounds have anti-inflammatory activities.

The main constituents of the petroleum ether extract of the bark of Maqian were 3(2H)-benzofuranone (26.173%), asarinin (6.484%) and (dimethoxymethyl)-3- methoxy-benzene (5.443%). 3(2H)-benzofuranone can affect the release of inflammatory mediators and reduce the measured levels of TNF-α at the concentration of 80 μM and showed a weak activity (Fig. 7E). Previous researches reported that benzofuran compounds, which were widespread in Rutaceae^36^, had shown multiple bioactivities, including anti-inflammatory^37^. Our study is consistent with these results. Asarinin, with a content of 6.484% in the petroleum ether extract of Maqian bark, is a furofuran type lignin with anti-inflammatory activity^38^. Nerolidol (2.451%), (+)-spathulenol (1.246%) and (−)-caryophyllene oxide (2.023%) are all the major constituents of the petroleum ether extract of Maqian bark, and previous studies showed that the essential oils of plants contain these compounds have anti-inflammatory activity^39^. It is inferred that the anti-inflammatory activity of the petroleum ether extract of the bark of Maqian by inhibiting NO, TNF-α and IL-1β production may be due to the presence of these compounds.

Compounds **1–18** (Table 2) were isolated from the ethyl acetate extract of Maqian bark. The main constituents of the ethyl acetate extract of the bark of Maqian were sitosterol (0.0159%), daucosterol (0.0144%), stigmasterol (0.0127%), hesperidin (0.0106%), decarine (0.0095%) and zanthoxyline dimethoxy derivative (0.0095%). Sitosterol, daucosterol and stigmasterol are widespread in plants. Many sterol analogs^40^ were previously reported to have anti-inflammatory activities by suppressing the secretion of inflammatory cytokines, such as TNF-α. Previous studies reported that sitosterol^41^, daucosterol^42^ and stigmasterol^43^ had shown anti-inflammatory activity. Alkaloids also have shown multiple bioactivities, including anti-inflammatory^44^. The anti-inflammatory effects of 9 compounds (Table 3) were evaluated for the inhibition of NO production in LPS-stimulated RAW264.7 cells. Among the compounds tested, compound **18,** decarine, showed the strongest anti-inflammatory activity with IC_50_ values of 48.43 μM (Table 3). Our studies also indicated anti-inflammatory activtity of decarine was realized by inhibiting TNF-α and IL-1β production (Figs 7F and 8F). Decarine reduced IL-6 and IL-8 production in TNF-α+IL-1β-induced Caco-2 cells (Fig. 9), which indicated anti-inflammatory activity on human colon cells and justified the use of Maqian as a remedy for colitis. It is inferred that the anti-inflammatory activity of the ethyl acetate extract of Maqian bark may be due to the presence of these compounds as decarine, sitosterol, daucosterol and stigmasterol mostly. Although a few compounds like hexadecanamide and adenosine showed pro-inflammatory properties (Table 3), they are not the major components and not affect the overall anti-inflammatory activity of the extract.

NO, IL-1β, IL-6, IL-8 and TNF-α are inflammatory mediators produced from nuclear factor kappa B (NF-κB) pathway. The inhibition of NO, IL-1β and TNF-α production in LPS-treated macrophages indicated that Maqian extracts and compounds might display anti-inflammatory activities through the inhibition of the NF-κB pathway. Decarine, the major anti-inflammatory compound of Maqian, is an isoquinoline alkaloid with a structure similar to berberine, which is a known anti-inflammatory compound^45^. Decarine showed the inhibition of NO, IL-1β, IL-6, IL-8 and TNF-α production as berberine, which indicates that decarine might exert anti-inflammation through the suppression of the NF-κB pathway too.

## Conclusions

In this study, the chemical constituents of the petroleum ether extract and the ethyl acetate extract, and the anti-inflammatory of the bark of Maqian (*Zanthoxylum myriacanthum* var. *pubescens*) are reported for the first time. 44 compounds in the petroleum ether extract were identified with GC-MS and 18 compounds in the ethyl acetate extract were isolated by phytochemical methods and identified by chemical and spectral analyses. All extracts show anti-inflammatory activity. The petroleum ether and ethyl acetate extracts have potent anti-inflammatory activity by decreasing NO, TNF-α and IL-1β production in LPS-induced RAW264.7 and THP-1 cells. 3(2H)-benzofuranone show a certain extent of anti-inflammatory activity on inhibiting NO and TNF-α release. Decarine, an alkaloid isolated and identified from the ethyl acetate extract, has anti-inflammatory activities on inhibiting the production of NO, TNF-α and IL-1β in LPS-treated macrophages, and inhibiting the production of IL-6 and IL-8 in Caco-2 cells induced by IL-1β and TNF-α. The IC_50_ values of decarine by inhibiting the production of NO are 48.43 μM on RAW264.7 cells. The anti-inflammatory activity supported the recorded medicinal use of Maqian for enteritis, pediatric hepatitis and colitis treatments in Xishuangbanna, China. These results provided a theoretical and material basis for the development and utilization of Maqian bark, a unilateral Dai medicine, and contributed to the standardization of Dai medicine.

## Additional Information

**How to cite this article**: Zhang, H.-l. *et al*. Chemical constituents and anti-inflammatory activities of Maqian (*Zanthoxylum myriacanthum* var. *pubescens*) bark extracts. *Sci. Rep.* **7**, 45805; doi: 10.1038/srep45805 (2017).

**Publisher's note:** Springer Nature remains neutral with regard to jurisdictional claims in published maps and institutional affiliations.

## Figures and Tables

**Table 1:** Chemical composition of petroleum ether extract from *Zanthoxylum myriacanthum* var. *pubescens* bark.

#	Compound name	RT(min)	Content
1	Hexanoic acid	12.502	0.51%
2	R(+)-Limonene	13.362	0.16%
3	Bornyl acetate	22.673	1.25%
4	2-Undecanone	22.887	0.90%
5	5-Pentyl-2(5H)-furanone	24.276	0.20%
6	(+)-Cycloisosativene	25.035	0.20%
7	alpha-Cubebene	25.264	0.20%
8	beta-Caryophyllene	26.461	0.92%
9	(+)-Aromadendrene	26.979	0.89%
10	1H-Cycloprop[e]azulene	27.54	0.26%
11	2-Tridecanone	28.331	1.35%
12	Nerolidol	29.03	2.45%
13	(+)-Spathulenol	30.462	1.25%
14	(−)-Caryophyllene oxide	30.575	2.02%
15	Hexadecane	30.799	0.46%
16	2,5,9-Trimethylcycloundeca-4,8-dienone	31.205	0.47%
17	Selin-11-en-4-.alpha.-ol	32.215	0.43%
18	4-Bromo-1-naphthalenamine	32.946	0.48%
19	7-Acetyl-2-hydroxy-2-methyl-5-isopropylbicyclo[4.3.0]nonane	34.068	0.51%
20	Platambin	36.606	0.50%
21	3,4-Dimethoxyphenethyl isothiocyanate	37.033	0.54%
22	Hexadecanoic acid, methyl ester	37.781	1.13%
23	Hexadecanoic acid	38.663	4.38%
24	2-(4-Hydroxyphenyl)-3-ethylindene	39.875	0.43%
25	9,12-Octadecadienoic acid, methyl ester	40.997	1.06%
26	9-Octadecenoic acid (z)-methyl ester	41.109	0.78%
27	Octadecanoic acid, methyl ester	41.563	0.21%
28	9,12-Octadecadienoic acid	41.927	4.12%
29	9-Octadecenoic acid	42.007	2.14%
30	Octadecanoic acid	42.277	0.59%
31	Tricosane	44.55	0.16%
32	3(2H)-Benzofuranone	45.816	26.17%
33	Tetracosane	46.195	0.24%
34	(Dimethoxymethyl)-3-methoxy-benzene	47.119	5.44%
35	Pentacosane	47.771	0.49%
36	Hexacosane	49.299	0.22%
37	1-Hydroxy-3,6-dimethoxy-8-methyl-xanthen-9-one	50.485	0.17%
38	Heptacosane	50.923	0.20%
39	Asarinin	63.579	6.48%
40	Sesamin	63.787	0.48%
41	Fargsin	65.705	3.04%
42	Campesterol	67.212	0.40%
43	Stigmast-5-en-3-ol	72.18	0.67%
44	Norchelerythrine	77.843	0.63%

RT: retention time.

**Table 2:** Chemical composition of ethyl acetate extract from *Zanthoxylum myriacanthum* var. *pubescens* bark.

	Compound name	amount	content
	ethyl acetate extract	473000 mg	100%
1	1-Bromo-7-methyl-(7 *S*)-nonadecane^20^	6 mg	0.0013%
2	Hexadecanamide^21^	20 mg	0.0042%
3	Machilusmarin^22^	15 mg	0.0032%
4	Dibutylphthalate^23^	2 mg	0.0004%
5	Salicylic acid^24^	8 mg	0.0017%
6	p-Hydroxybenic acid^25^	6 mg	0.0013%
7	Adenosine^26^	8 mg	0.0017%
8	Alhagidin^27^	40 mg	0.0085%
9	Quercetin-3-*O*-α-L-arabinopyranoside^28^	4 mg	0.0008%
10	Hesperidin (hesperetin-7-*O*-rutinoside)^29^	50 mg	0.0106%
11	Rhoifoline B^30^	5 mg	0.0011%
12	Zanthoxyline dimethoxy derivative^31^	45 mg	0.0095%
13	N-Nortidine^32^	5 mg	0.0011%
14	Nitidine^33^	10 mg	0.0021%
15	Sitosterol	75 mg	0.0159%
16	Stigmasterol	60 mg	0.0127%
17	Daucosterol	68 mg	0.0144%
18	Decarine^34^	45 mg	0.0095%

**Table 3:** The inhibition of N.O production by major compounds from *Zanthoxylum myriacanthum* var. *pubescens* bark.

	Compound name	IC_50_(μM)	Inhibition of NO production at concentration of 50 μΜ
2	hexadecanamide	>50	−33.46% ± 2.21
7	adenosine	>50	−4.30% ± 3.04
8	alhagidin	>50	6.09% ± 0.62
10	hesperidin (hesperetin-7-*O*-rutinoside)	>50	0.54% ± 0.76
11	rhoifoline B	>50	12.13% ± 2.30
12	zanthoxyline dimethoxy derivative	>50	8.09% ± 2.30
13	N-nitidine	>50	2.21% ± 1.69
14	nitidine	>50	19.36% ± 1.08
18	decarine	48.43	51.06% ± 0.00
	3(2H)-benzofuranone	>50	11.19% ± 4.90
